# Brazilian Cardiorespiratory Fitness Classification Based on Maximum Oxygen Consumption

**DOI:** 10.5935/abc.20160070

**Published:** 2016-05

**Authors:** Artur Haddad Herdy, Ananda Caixeta

**Affiliations:** 1 Instituto de Cardiologia de Santa Catarina, Florianópolis, SC - Brazil; 2 Clínica Cardiosport, Florianópolis, SC - Brazil; 3 Universidade do Sul de Santa Catarina, Florianópolis, SC - Brazil

**Keywords:** Respiratory Function Tests, Exercise, Exercise Test, Oxygen Consumption

## Abstract

**Background:**

Cardiopulmonary exercise test (CPET) is the most complete tool available to assess functional aerobic capacity (FAC). Maximum oxygen consumption (VO_2_ max), an important biomarker, reflects the real FAC.

**Objective:**

To develop a cardiorespiratory fitness (CRF) classification based on VO_2_ max in a Brazilian sample of healthy and physically active individuals of both sexes.

**Methods:**

We selected 2837 CEPT from 2837 individuals aged 15 to 74 years, distributed as follows: G1 (15 to 24); G2 (25 to 34); G3 (35 to 44); G4 (45 to 54); G5 (55 to 64) and G6 (65 to 74). Good CRF was the mean VO_2_ max obtained for each group, generating the following subclassification: Very Low (VL): VO_2_ < 50% of the mean; Low (L): 50% - 80%; Fair (F): 80% - 95%; Good (G): 95% -105%; Excellent (E) > 105%.

**Results:**

**Table:** 

Men	VL < 50%	L 50-80%	F 80-95%	G 95-105%	E > 105%
G1	< 25.30	25.30-40.48	40.49-48.07	48.08-53.13	> 53.13
G2	< 23.70	23.70-37.92	37.93-45.03	45.04-49.77	> 49.77
G3	< 22.70	22.70-36.32	36.33-43.13	43.14-47.67	> 47.67
G4	< 20.25	20.25-32.40	32.41-38.47	38.48-42.52	> 42.52
G5	< 17.54	17.65-28.24	28.25-33.53	33.54-37.06	> 37.06
G6	< 15	15.00-24.00	24.01-28.50	28.51-31.50	> 31.50
**Women**					
G1	< 19.45	19.45-31.12	31.13-36.95	36.96-40.84	> 40.85
G2	< 19.05	19.05-30.48	30.49-36.19	36.20-40.00	> 40.01
G3	< 17.45	17.45-27.92	27.93-33.15	33.16-34.08	> 34.09
G4	< 15.55	15.55-24.88	24.89-29.54	29.55-32.65	> 32.66
G5	< 14.30	14.30-22.88	22.89-27.17	27.18-30.03	> 30.04
G6	< 12.55	12.55-20.08	20.09-23.84	23.85-26.35	> 26.36

**Conclusions:**

This chart stratifies VO_2_ max measured on a treadmill in a robust Brazilian sample and can be used as an alternative for the real functional evaluation of physically and healthy individuals stratified by age and sex.

## Introduction

Cardiopulmonary exercise test (CPET) is considered one of the most complete tools to assess functional aerobic capacity, because it provides an integrated assessment of response to exercise, involving the cardiovascular, pulmonary, hematopoietic, neurophysiological and skeletal muscle systems.^1^ In clinical practice, it has been widely used to assess cardiac and pulmonary diseases, to stratify the risk of patients with heart failure, and to optimize the prescription of physical exercise.^2-5^ In Brazil, CPET is preferably performed on a treadmill, but, in many countries, a cycle ergometer is preferred. Maximum oxygen consumption (VO_2_ max) reflects the individual's maximum capacity to absorb, transport and consume oxygen.^2^ The major determinants of normal VO_2_ max are: genetic factors, muscle mass amount, age, sex and body weight.^1,2^ In practice, VO_2_ max is considered to be equivalent to the highest VO_2_ value obtained in peak exertion, which is usually used to classify cardiorespiratory fitness (CRF) in a population. In this study, for practical purposes, we named VO_2_ peak, which was actually measured, VO_2_ max.

Few studies have provided reference CRF charts for populations, and it is yet to be clarified whether the existing classifications can be extrapolated to other populations. Most published studies have been based on small samples, and the profiles of the populations studied have significantly differed.^6,7^ The CRF classification charts most used in Brazil are as follows: that of the American Heart Association (AHA), published in 1972 (Table 1), and that by Cooper, of 1987. Brazil does not have a solid and widely used CRF classification for CPET; therefore, this study proposes a classification based on Brazilian population data. Such data, resulting from a recently published study, were used as reference for CPET on a treadmill (ramp protocol) for sedentary and physically active men and women.^8^

**Table 1:** American Heart Association Cardiorespiratory Fitness Chart based on maximum oxygen consumption (VO_2_ max – mL/kg.min) – 1972

Men	Very Low	Low	Fair	Good	Excellent
**Age group**					
20-29	< 25	25-33	34-42	43-52	≥ 53
30-39	< 23	23-30	31-38	39-48	≥ 49
40-49	< 20	20-26	27-35	36-44	≥ 45
50-59	< 18	18-24	25-33	34-42	≥ 43
60-69	< 16	16-22	23-30	31-40	≥ 41
**Women**	**Very Low**	**Low**	**Fair**	**Good**	**Excellent**
**Age group**					
20-29	< 24	24-30	31-37	38-48	≥ 49
30-39	< 20	20-27	28-33	34-44	≥ 45
40-49	< 17	17-23	24-30	31-41	≥ 42
50-59	< 15	15-20	21-27	28-37	≥ 38
60-69	< 13	13-17	18-23	24-34	≥ 35

## Methods

This study's sample comprised 9,250 CPET performed at a large cardiology referral center in southern Brazil.^8^ Based on a questionnaire completed during the test, individuals with the following characteristics were excluded from the study: any symptom suggesting disease or pathology; amateur or professional athletes; smokers; users of any medication; obese individuals (body mass index - BMI > 30); and tests with the ratio between the amount of carbon dioxide produced and of oxygen used (respiratory exchange ratio - RER) < 1.1. After applying the exclusion criteria, 3,922 CPET were identified, of which, 2,837 CPET, corresponding to healthy and active individuals, were selected. Those individuals, aged between 15 and 74 years, were of both sexes and different ethnicities, and practiced leisure-time aerobic physical activity for at least 30 minutes a day, three times a week.^8^

All exercise tests were conducted by cardiologists trained in ergometry and CPET by the Brazilian Society of Cardiology Department of Ergometry and Cardiovascular Rehabilitation. The tests were performed on a treadmill (Inbrasport - ATL™, Brazil, 1999, Software ErgoPC Elite Version 3.3.6.2, Micromed Brazil, 1999), using the ramp protocol. A mixing chamber gas analyzer (MetaLyzer II, Cortex™ - Leipzig, Germany, 2004) was used to collect the expired gases. For descriptive statistics, central trend measures, such as means, were used, in addition to dispersion measures (standard deviation). Excel software, Microsoft 2008, was used for statistical analyses and charts.

Participants, classified according to sex (female and male), were divided into six age groups between 15 and 74 years as follows: G1 (15 to 24 years); G2 (25 to 34 years); G3 (35 to 44 years); G4 (45 to 54 years); G5 (55 to 64 years); and G6 (65 to 74 years).

The CRF classification proposed in this study was based on 2,837 CPET performed in apparently healthy individuals. We arbitrarily adopted as "Good" CRF the mean VO_2_ max value expressed in mL.kg^-1^.min^-1^ obtained in each group, and, taking that value as a reference, we classified CRF as follows: "Very Low" (VO_2_ value < 50% of the mean); "low" (50-80%); "fair" (80-95%); "good" (95-105%); and "excellent" (> 105%).

To internally validate our proposed CRF classification, sedentary individuals of both sexes from the study population sample were assessed, according to previous publication.^8^

This study was approved by the Ethics Committee in Research of the Instituto de Cardiologia de Santa Catarina.

## Results

Tables 2 and 3 show the mean VO_2_ max values of the original population and the number of CPET performed, stratified by sex and age groups, of physically active and sedentary individuals. The VO_2_ max levels were higher in the active groups as compared to the sedentary ones, and men had greater VO_2_ max levels than women did. Tables 4 and 5 show our proposed CRF classification, with five different categories, stratified by sex and age group, of apparently healthy individuals. Table 6 shows the classification of the sedentary population (men and women) from the original sample, considering the new CRF chart proposed in this study. It is worth noting that the CRF of sedentary individuals is always classified as either fair or low.

**Table 2:** Distribution of the physically active and sedentary male population according to mean VO_2_ max (mL/kg.min) and age groups

Active men						
Age (years)	15 – 24	25 – 34	35 – 44	45 – 54	55 – 64	65 – 74
n = 1818	343	597	427	285	134	32
Mean VO_2_ max (mL/kg.min)	50.6 ± 7.3	47.4 ± 7.4	45.4 ± 6.8	40.5 ± 6.5	35.3 ± 6.2	30 ± 6.1
**Sedentary men**						
Age (years)	15 – 24	25 – 34	35 – 44	45 – 54	55 – 64	65 – 74
n = 570	85	188	157	100	30	10
Mean VO_2_ max (mL/kg.min)	47.4 ± 7.9	41.9 ± 7.2	39.9 ± 6.8	35.6 ± 7.7	30 ± 6.3	23.1 ± 6.3

**Table 3:** Distribution of the physically active and sedentary female population according to mean VO_2_ max (mL/kg.min) and age groups

Active women						
Age (years)	15 – 24	25 – 34	35 – 44	45 – 54	55 – 64	65 – 74
n = 1019	177	300	229	206	81	26
Mean VO_2_ max (mL/kg.min)	38.9 ± 5.7	38.1 ± 6.6	34.9 ± 5.9	31.1 ± 5.4	28.6 ± 6.1	25.1 ± 4.4
**Sedentary women**						
Age (years)	15 – 24	25 – 34	35 – 44	45 – 54	55 – 64	65 – 74
n = 515	85	149	108	108	40	25
Mean VO_2_ max (mL/kg.min)	35.6 ± 5.7	34.0 ± 4.8	30.0 ± 5.4	27.2 ± 5.0	23.9 ± 4.2	21.3 ± 3.4

**Table 4:** Classification of cardiorespiratory fitness based on maximum oxygen consumption (VO_2_ max – mL/kg.min) for the male sex

Age group (years)	Very Low	Low	Fair	Good	Excellent
15 – 24	< 25.30	25.30 – 40.48	40.49 – 48.07	48.08 – 53.13	> 53.13
25 – 34	< 23.70	23.70 – 37.92	37.93 – 45.03	45.04 – 49.77	> 49.77
35 – 44	< 22.70	22.70 – 36.32	36.33 – 43.13	43.14 – 47.67	> 47.67
45 – 54	< 20.25	20.25 – 32.40	32.41 – 38.47	38.48 – 42.52	> 42.52
55 – 64	< 17.54	17.65 – 28.24	28.25 – 33.53	33.54 – 37.06	> 37.06
65 – 74	< 15	15.00 – 24.00	24.01 – 28.50	28.51 – 31.50	> 31.50

**Table 5:** Classification of cardiorespiratory fitness based on maximum oxygen consumption (VO_2_ max – mL/kg.min) for the female sex

Age group (years)	Very Low	Low	Fair	Good	Excellent
15 – 24	< 19.45	19.45 – 31.12	31.13 – 36.95	36.96 – 40.84	> 40.85
25 – 34	< 19.05	19.05 – 30.48	30.49 – 36.19	36.20 – 40.00	> 40.01
35 – 44	< 17.45	17.45 – 27.92	27.93 – 33.15	33.16 – 34.08	> 34.09
45 – 54	< 15.55	15.55 – 24.88	24.89 – 29.54	29.55 – 32.65	> 32.66
55 – 64	< 14.30	14.30 - 22.88	22.89 – 27.17	27.18 – 30.03	> 30.04
65 – 74	< 12.55	12.55 – 20.08	20.09 – 23.84	23.85 – 26.35	> 26.36

**Table 6:** Classification of cardiorespiratory fitness based on maximum oxygen consumption (VO_2_ max – mL/kg.min) of the male and female sedentary population from the original study and according to the new cardiorespiratory fitness chart proposed in this study

Men					
**Age group (years)**	**Very Low**	**Low**	**Fair**	**Good**	**Excellent**
15 – 24					VO_2_ = 47.4
25 – 34					VO_2_ = 41.9
35 – 44					VO_2_ = 39.9
45 – 54					VO_2_ = 35.6
55 – 64					VO_2_ = 30.0
65 – 74		VO_2_ = 23.2			
**Women**					
15 – 24					VO_2_ = 35.6
25 – 34					VO_2_ = 34.0
35 – 44					VO_2_ = 30.0
45 – 54					VO_2_ = 27.2
55 – 64					VO_2_ = 23.9
65 – 74					VO_2_ = 21.2

**Figure 1:**
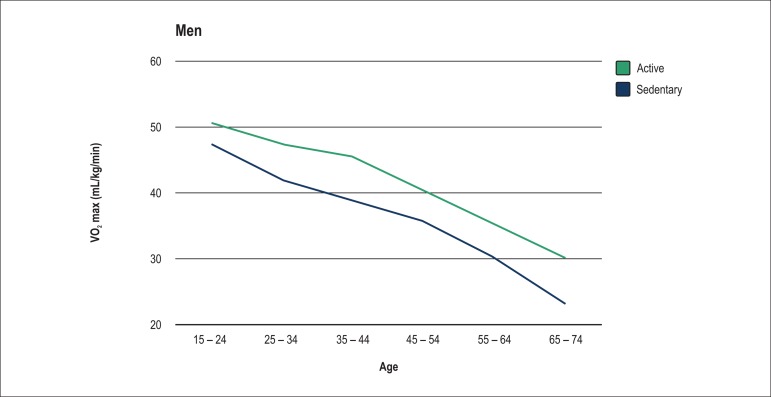
Behavior of maximum oxygen consumption (VO_2_ max – mL/kg.min) throughout the years in men.

**Figure 2:**
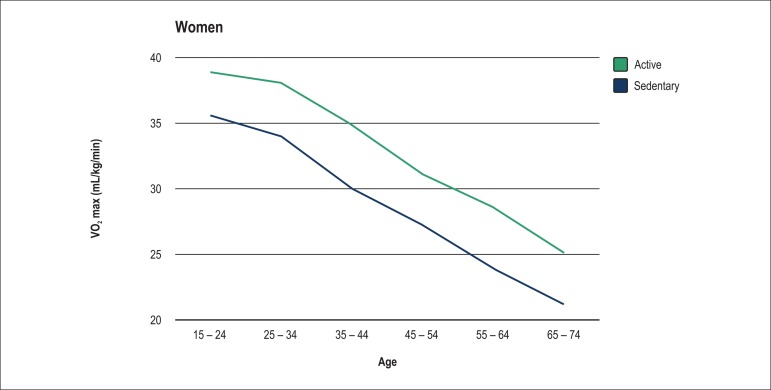
Behavior of maximum oxygen consumption (VO_2_ max – mL/kg.min) throughout the years in women.

As expected, VO_2_ max levels dropped throughout the age groups for both sexes (Figures 1 and 2).

## Discussion

We elaborated a CRF classification chart based on VO_2_ max levels measured during CPET (ramp protocol) performed on an ergometric treadmill, to more accurately classify a solid Brazilian sample of healthy and physically active individuals of both sexes. We chose to base our analysis on data of physically active individuals, who would provide CRF in the "good" category, corresponding to mean CRF values. Not using data of sedentary individuals allowed us to validate our proposed CRF classification chart, observing in which category sedentary individuals would fit.

According to our CRF classification chart, we confirmed that the CRF of active men is higher than that of active women of the same age group, and, for both sexes, active individuals had a better CRF as compared to sedentary ones. According to Nunes et al.,^7^ mean VO_2_ max values of women are lower than those of men, the mean VO_2_ max values of the former corresponding to only 70% of those of the latter. The present study showed a mean VO_2_ max of women corresponding to 76% to 83% of the mean VO_2_ max of men of the same age group.

Sedentary individuals not only had a lower VO_2_ max as compared to physically active ones, but also a twice higher decrease in VO_2_ max as age advanced.^9,10^ Regular exercise practice reduces the VO_2_ max rate of decrease as compared to a sedentary lifestyle,^11^ and, the greater the VO_2_, the greater the protection against cardiovascular events. An increase in aerobic capacity is associated with an increase in survival, as reported by Myers et al.,^12^ who have demonstrated a significant increase in the relative risk of death from any cause as functional capacity decreased, regardless of the risk factors involved. In addition, those authors have reported a 12%-increase in survival for each 1-MET increase in the CRF level.^12^

Most CRF classification charts used in clinical practice have been elaborated in other countries and have not been validated for the Brazilian population. Extrapolating those classifications to the Brazilian population can lead to relevant discrepancies. Belli et al.^13^ have shown significant discrepancies when comparing international charts with Brazilian data. Nunes et al.^7^ have classified CRF into percentiles, similarly to Cooper et al., and have observed a difference in VO_2_ max when comparing the two charts.

VO_2_ max depends on a frequent and constant physical activity and can be enhanced with treinos.^14^ However, despite the volume or intensity of the workout raise VO2 max by 10 to 30%, there is also an important genetic influence. Research has shown that genetic inheritance is the main responsible for max VO2 each individual and may be responsible for up to 25% to 50% of the variation in the values of VO_2_ max, ie, alone accounts for almost half of ACR.^15^

VO_2_ max can be measured directly by analyzing the gases expired during CPET, or indirectly, by using calculations. Although some prediction equations provide an acceptable association with values obtained via direct measurements, the difference varies, depending on the population studied. The error for one certain individual can be extremely high, ranging from 15% to 20% in some studies, and can even reach or exceed 30%, a high margin of error, considering other measurements in the biological area^16^.

According to data obtained in this study, VO_2_ max drops with age. That drop in women varies less from one age group to the other as compared to that in men. We observed a higher drop in VO_2_ max among active women from group 3 to group 4, with a mean of 0.38 mL.kg^-1^.min^-1^ per year. Among sedentary women, that drop was sharper from group 2 to group 3, with a mean of 0.4 mL.kg^-1^.min^-1^ per year. Among both active and sedentary men, however, the VO_2_ max drop was more marked from group 5 to group 6, with a mean of 0.53 mL.kg^-1^.min^-1^ per year among active men, and of 0.69 mL.kg^-1^.min^-1^ per year among sedentary men. Nunes et al.^7^ have shown a VO_2_ max drop of 0.4 mL.kg^-1^.min^-1^ per year among men aged 20 to 60 years. Belli et al.,^13^ using indirect VO_2_ max measurement, have evidenced a drop of 20% to 25% per decade in mean VO_2_ max from the age of 50 years onward, that drop being sharper after the age of 60 years. An approximate drop in VO_2_ max of 0.4 mL.kg^-1^.min^-1^ per year is estimated to occur from the age of 25 years onward, and that VO_2_ max decline is twice greater in sedentary individuals as compared to physically active ones.^8,9^

We used the new CRF classification chart to classify sedentary individuals undergoing CPET under the same conditions of the physically active ones from the original population. This would allow us to validate our proposed classification, considering how the VO_2_ max values of those individuals would fit. Differently from the studies estimating VO_2_ max indirectly, the direct measurement of VO_2_ max shows that CRF in sedentary individuals is classified, at the most, as fair, regardless of age and sex (Table 6). From the practical viewpoint, sedentary individuals have decreased tolerance to exertion, and, thus, physical exercise prescription to active and sedentary individuals should differ.^17^

The CRF chart by Cooper^18^ and that of the AHA^19^ (Table 1) are the most commonly used tools to classify CRF in CPET programs in Brazil. However, the literature lacks data concerning sampling methods and sample types used to elaborate the AHA chart. Therefore, the comparison of data obtained in this study with the AHA chart is limited. Our classification comprises a wider age range, from 15 to 74 years, as compared to that of the AHA (20 to 69 years). The VO_2_ max analysis in both charts evidences, in younger age groups, very similar VO_2_ max values. However, in the other age groups, a greater difference is observed between our data and the VO_2_ max values of the AHA chart.

Most CRF charts published so far have been elaborated with CPET performed on a cycle ergometer. The VO_2_ max obtained in tests performed on a treadmill, as opposed to those performed on a bicycle, is approximately 5% to 17% higher (mean of 8%).^20,21^ The difference is attributed to the amount of active muscle mass involved in the test, which is greater for the inclined treadmill. Another important factor relates to the pedaling effect, which causes localized muscle fatigue by using the large muscle groups of the thigh, and that fatigue can occur before maximum exertion is imposed to the circulatory and respiratory systems, generating a lower VO_2_ max.^2^

In our study sample, the age range was wide, including adolescents older than 15 years. We believe that from that age on, individuals already have muscle maturation and performance close to those of young adults under the age of 25 years.^22,23^ The classification chart proposed should be assessed as an instrument to predict risk for morbidity and mortality, according to each individual's functional profile. Further studies are required.

This study has limitations, such as the lack of standardization of ramp protocols. Individuals classified as physically active practiced different types of activities and sports, making the comparison of the results in different populations difficult. Further studies are required, using the same intensities and inclinations in the protocol ramp and with individuals practicing the same type of aerobic exercise, because that would improve the analysis and comparison of the results. Individuals with hypertension, diabetes or dyslipidemia, those on any type of medication, and those with a BMI greater than 30 (obese) were excluded, making the applicability of that classification in those subgroups uncertain. The Brazilian population is known to be diversified, and, in southern Brazil, the European colonization predominates (smaller percentage of Afrodescendant and Native individuals, as compared to other Brazilian regions). New studies should be developed, including different ethnicities and individuals from other Brazilian regions, aiming at comparing with the classification proposed to verify whether the values differ.

## Conclusion

This is one of the few Brazilian studies to propose a CRF chart with data extracted from a robust population sample, and based on VO_2_ max measured via CPET on a treadmill. These data can be used for functional capacity classification according to sex and age group and considering different risk profiles.
